# Assessing the impact of the COVID-19 pandemic on uptake of HIV treatment in Bandung and Yogyakarta, Indonesia: A retrospective cohort study

**DOI:** 10.1371/journal.pgph.0005666

**Published:** 2025-12-23

**Authors:** Yusuf Ari Mashuri, David Boettiger, Srila Nirmithya Salita Negara, Siska Dian Wahyuningtias, Ari Probandari, Marco Liverani, Luh Putu Lila Wulandari, Riris Andono Ahmad, Hasbullah Thabrany, Nasser Fardousi, John Kaldor, Yanri Wijayanti Subronto, Virginia Wiseman

**Affiliations:** 1 Center for Tropical Medicine, Faculty of Medicine, Public Health and Nursing, Universitas Gadjah Mada, Yogyakarta, Indonesia; 2 Faculty of Medicine, Universitas Sebelas Maret, Surakarta, Indonesia; 3 The Kirby Institute, University of New South Wales, Sydney, Australia; 4 Department of Global Health and Development, London School of Hygiene and Tropical Medicine, London, United Kingdom; 5 School of Tropical Medicine and Global Health, Nagasaki University, Nagasaki, Japan; 6 Department of Biostatistics, Epidemiology, and Population Health, Faculty of Medicine, Public Health and Nursing, Universitas Gadjah Mada, Yogyakarta, Indonesia; 7 ThinkWell.Global, Jakarta, Indonesia; 8 Division of Tropical Medicine and Infectious Diseases, Department of Internal Medicine, Faculty of Medicine, Public Health, and Nursing, Universitas Gadjah Mada/Dr. Sardjito General Hospital, Yogyakarta, Indonesia; Monash University Indonesia, INDONESIA

## Abstract

COVID-19 pandemic known to affect health service deliveries including for HIV care support and treatment. In this retrospective study involving 2,780 people living with HIV (PLHIV), we evaluated impact of COVID-19 pandemic by comparing the proportion of PLHIV linked to care, started antiretroviral therapy (ART), retained in care (within the first 3 months of treatment), and adhered to ART (within the first 3 months of treatment) between the pre-pandemic period (2018–2019) and pandemic period (2020–2021) in Yogyakarta and Bandung, Indonesia. Our study showed that during the pandemic period the number of PLHIV linked to care was 18% lower (1,529 vs 1,251) and those retained in care was significantly lower (59.6% vs 53.3%, p = 0.0009) than the pre-pandemic period. Whereas, proportion in ART initiation (79.6% vs 78.3%, p = 0.3892) and ART adherence (50.0% vs 46.8%, p = 0.1010) were not statistically different. Multivariate analysis showed that ART initiation (aOR = 1.00, p = 0.996) nor retention in care (aOR = 0.90, p = 0.344) were not significantly different between two period cohorts. Adherence for the first three months of treatment, however, was significantly higher in the pandemic cohort (aOR = 1.53, p = 0.009). In the subgroup analysis, older PLHIV and those attending hospitals (tertiary versus primary care clinics) were significantly less likely to initiate ART, be retained in care, or adhere to ART. This study provides evidence of the impact of the COVID-19 pandemic on several characteristics of the HIV treatment cascade such as lower number of linkage to- and retention in care, lower number of older PLHIV, and attendance to tertiary care (hospital). General and HIV-specific mitigation strategies should be designed to minimise pandemic related disruptions and to support the continuity of HIV care to face possible future health crises.

## Introduction

The COVID-19 pandemic has had a massive impact on public health services worldwide. In Indonesia, there were 6,829,221 cases of COVID-19, and 162,063 COVID-19 deaths recorded between 2020–2024 [1]. Most cases were on Java Island [2]. In 2024, Indonesia reported an estimated 570,000 people living with HIV (PLHIV), more than in any other country in Southeast Asia [3,4]. HIV/AIDS cases were most prevalent on Java Island, particularly in East Java, West Java, and Central Java [5]. In 2024, only 60% of PLHIV were aware of their status; among them, 67% were on antiretroviral therapy (ART), 59% of those on ART had undergone viral load testing, and just 56% achieved viral suppression [5].

Prior to the pandemic, HIV services in Indonesia routinely delivered through a network of Comprehensive Care and Support for Treatment (CST) facilities via monthly in-person visits, during which patients received one-month ART supplies, adherence counselling, and laboratory monitoring [6]. However, during the early stages of the COVID-19 pandemic, concerns were quickly raised about the potential disruptions to HIV treatment and care and the increased risk of PLHIV developing severe illness [7–11].

In an effort to control the pandemic, the Indonesian government imposed wide scale mobility restrictions and reprioritized health care funding towards COVID-19, resulting in reduced access to many other core health services [12,13]. Given that much of Indonesia’s HIV program is supported by international donors (28.8%), including the Global Fund, this heavy reliance on external funding may have further exacerbated the health system’s vulnerability during the COVID-19 crisis, particularly on key population outreach and peer support [14]. Evidence from a range of low- and middle-income countries (LMIC) showed that these types of restrictions had the potential to undermine access to HIV treatment, a central element of HIV control strategies globally, by disrupting medicine/diagnostic supplies, diverting staff from testing, counselling, and outreach services [15–17]. To mitigate these effects, the Ministry of Health issued pandemic-specific guidelines in 2020, including multi-month ART dispensing (2–3 months), triage, and COVID-19 symptom screening at clinics [18]. Despite these efforts, increased fear of COVID-19 in the community and rising levels of unemployment and poverty threatened access to HIV care [19], increasing the risk of HIV treatment failure, disease relapse/progression, and transmission of infection [20,21].

Several investigations reported on the impact of the COVID-19 pandemic on services for HIV-related key populations in Indonesia [22–24]. However, none of these directly measured the impact of the COVID-19 pandemic on the uptake and continuity of HIV treatment using well-characterised clinical cohorts, for Indonesia or any other South-East Asia country [25,26]. Our study sought to address this gap by assessing the impact of the pandemic on people enrolled in HIV treatment in two cities in Indonesia experiencing a high burden from COVID-19.

## Methods

### Ethics statement

The DOMINO study was approved by the ethics committees of Universitas Gadjah Mada Yogyakarta (No KE/FK/1410/EC/2021), the London School of Hygiene and Tropical Medicine (No 22829), and the University of New South Wales (No HC200989). All three ethics committees approved the waiving of consent for patient inclusion in our cohort study on the basis that health facilities contributed de-identified medical records data to protect privacy.

### Study design

This study was part of the DOMINO study, which sought to measure the effects of the COVID-19 pandemic on tuberculosis and HIV care in Indonesia. Observational retrospective cohort data on PLHIV linked to care were collected from 2018 – 2021. For this analysis, we compare two datasets from 2018-2019 (representing the pre-COVID-19 pandemic period) and from 2020-2021 (representing the during COVID-19 pandemic period). Data collection was conducted from 01 December 2021 until 01 December 2022.

### Study setting

The study was conducted in two major cities on Java Island, i.e., Bandung and Yogyakarta. These two cities were selected based on their high number of COVID-19 cases and heavy HIV burden [27,28]. Between 2020 and 2021, Bandung reported 99,324 COVID-19 cases and 1,423 COVID-19 deaths [29]. In 2021, there were 2,397 people known to be living with HIV [27]. For the same time period, Yogyakarta reported 35,763 COVID-19 cases, 1,164 COVID-19 deaths, and 1,421 HIV cases [28,30]. The sites of Bandung and Yogyakarta were selected to reflect conditions in larger Indonesian cities and the findings are expected to be applicable to other cities beyond just the most affected areas [31,32]. Feasibility factors also influenced site selection, including our ability to effectively engage with existing staff working in city health offices and health facilities.

Indonesia’s health system comprises both public and private providers with decentralized administration. Private healthcare includes hospitals and clinics run by non-profit organizations, for-profit entities, and individual practitioners who may work both in public and private sectors. Public healthcare services are stratified between primary health care centres (also known as *Pusat Kesehatan Masyarakat* or *Puskesmas*), and hospitals [33]. *Puskesmas* serve as primary points of contact, offering a wide range of services including curative, rehabilitative, preventive, and promotive care both onsite and through community outreach programs. In contrast, hospitals provide specialised and tertiary care [33].

Services for PLHIV are administered through a network of Comprehensive Care and Support for Treatment (CST) facilities, which deliver antiretroviral therapy (ART). In Bandung, there are eleven government CST and five private CST facilities, the corresponding numbers for Yogyakarta being seven government CST and three private CST facilities [27,30]. However, there are no formal national criteria determining whether PLHIV receive care at hospitals or primary health centres. Service utilization depends on clinical condition of the patient, the availability and capacity of local facilities to provide CST services, including one-stop testing and treatment. Patients with HIV clinical stage 3 or 4 required specialized care and laboratory equipment [34].

At CST facilities, data on PLHIV who have been linked to care but have not yet initiated ART are recorded in the pre-ART register, while those who have started ART are recorded in the ART register. National guidelines recommend same-day or seven-days at the latest of ART initiation upon HIV diagnosis for asymptomatic patient. However, ART initiation also depends on clinical factors and/or patient readiness [35]. Health facilities upload their pre-ART and ART data every month to the national HIV/AIDS Information System (SIHA/*Sistem Informasi HIV/AIDS*) developed by the Indonesian Ministry of Health in 2012 [36]. This data collection process remained consistent during the entire study period.

### Study procedures

Data was collected from the pre-ART and ART registers in fifteen public CST health facilities, including nine *Puskesmas*, four hospitals, and two private clinics, with four facilities located in Yogyakarta and eleven in Bandung. All PLHIV enrolled in care from January 2018 to December 2021 were included. Data were extracted from all participating facilities. Prior to analysis, all data were verified together with health facilities and the City Health Offices, and pre-ART and ART data were merged based on the national registration number of each PLHIV. We included all individuals recorded in the SIHA registry with no exclusion criteria applied. All research activities were undertaken with the permission from the City Health Offices.

### Data analysis

Data were analysed using Stata 17 (StataCorp, College Station, TX, USA). For each study period (i.e., pre-COVID-19 and during COVID-19), we determined the number of PLHIV linked to care, started on ART, retained in care, and adherent to ART. Although viral suppression is the preferred indicator of treatment success, most PLHIV in Indonesia do not receive viral load (VL) testing due to the limited availability of testing devices [37]. Similarly, CD4 testing remains inconsistent, with only 53% of PLHIV receiving baseline CD4 testing [38]. As a result, we were unable to include viral suppression and CD4 count in our treatment cascade analysis. We have used the term ‘linked to care’ to define clients diagnosed with HIV who successfully commenced treatment. ‘Started ART’ includes any clients diagnosed with HIV who entered treatment services and began ART based on information in the SIHA registry for each time period (2018–2019 and 2020–2021). ‘Retained in care’ includes PLHIV that started ART and had at least two outpatient visits during the first 3 months of ART. ‘Adherent to ART’ includes PLHIV retained in care with a documented medication adherence rate of at least 95% during the first three months of treatment. Medication adherence is based on ART refill timeliness, defined as within 30 days of the scheduled date [39,40]. PLHIV linked to care in the last three months of the ‘pre-COVID-19’ study period (i.e., October-December 2019) or ‘during the COVID-19’ study period (i.e., October-December 2021) were excluded from our analyses as they could not meet our definition of ‘retained in care’ or ‘adherent to ART’. Binomial confidence intervals were calculated for the proportion of individuals starting ART, retained in care, and adherent to ART. Logistic regression was used to evaluate the impact of COVID-19 at each step in our HIV care cascade and when adjusting for demographic and health facility characteristics including: age; sex; education; employment status; marital status; HIV key populations (men who have sex with men, female sex workers, people who inject drugs, transgender women and non-key population); and facility type (*Puskesmas* and private clinic, and hospital). In our final multivariate model, all covariates were retained regardless of statistical significance. Individuals with missing covariate data were retained in all analyses by assigning them to a missing category for the covariate. We also performed interaction analyses to assess the impact of COVID-19 among study sub-populations defined by the above-mentioned demographic and health facility characteristics.

## Results

### HIV treatment cascade – whole study population

The study included 2,780 PLHIV enrolled in care. Of those, 1,529 were linked to care pre-COVID-19 pandemic and 1,251 were linked to care during the pandemic. Table 1 shows that each cohort’s demographic and clinical characteristics were broadly consistent.

**Table 1:** Characteristics of PLHIV linked to care pre-COVID-19 and during the COVID-19 pandemic.

	Pre-COVID-19 (n = 1,529), %	During-COVID-19 (n = 1,251), %
**Age (years)**		
Mean (SD)	29.9 (8.6)	29.5 (87)
Median (IQR)	28 (23 - 34)	27 (23 - 34)
**Age group**		
<16	1 (0.1)	2 (0.2)
16-19	50 (3.3)	60 (4.8)
20-29	834 (54.5)	687 (54.9)
30-39	429 (28.1)	339 (27.1)
40-59	204 (13.3)	150 (12.0)
>60	8 (0.5)	10 (0.8)
Missing data	3 (0.2)	3 (0.2)
**Sex**		
Male	1343 (87.8)	1122 (89.7)
Female	186 (12.2)	129 (10.3)
**Highest level of education**		
Primary school or less	67 (4.4)	41 (3.3)
High school	902 (59.0)	736 (58.8)
University or diploma	460 (30.1)	260 (20.8)
Missing data	100 (6.5)	214 (17.1)
**Employment**		
Employed or self-employed	1,100 (71.9)	806 (64.4)
Unemployed or student	332 (21.7)	231 (18.5)
Missing data	97 (6.3)	214 (17.1)
**Marital status**		
Married	312 (20.4)	185 (14.8)
Not Married	1,052 (68.8)	656 (52.4)
Divorced	84 (5.5)	44 (3.5)
Missing Data	81 (5.3)	366 (29.3)
**Population group**		
Men Who Have Sex with Men	947 (61.9)	756 (60.4)
Female Sex Workers	159 (10.4)	91 (7.3)
People Who Inject Drugs	36 (2.4)	32 (2.6)
Transgender Women	7 (0.5)	3 (0.2)
Non-Key Population	358 (23.4)	358 (28.6)
Missing Data	22 (1.4)	11 (0.9)
**Pregnancy status (Female only; n = 315)**		
Pregnant	8 (4.3)	6 (4.7)
Not Pregnant	114 (61.3)	52 (40.3)
Missing Data	64 (34.4)	71 (55.0)
**Clinical stage**		
Asymptomatic	802 (52.5)	664 (53.1)
Mild symptoms	122 (8.0)	63 (5.0)
Severe symptoms	130 (8.5)	64 (5.1)
AIDS	23 (1.5)	13 (1.0)
Missing Data	452 (29.6)	447 (35.7)
**Health Facilities**		
*Puskesmas* and Private Clinic	926 (60.6)	911 (72.8)
Hospital	603 (39.4)	340 (27.2)

**Fig 1:**
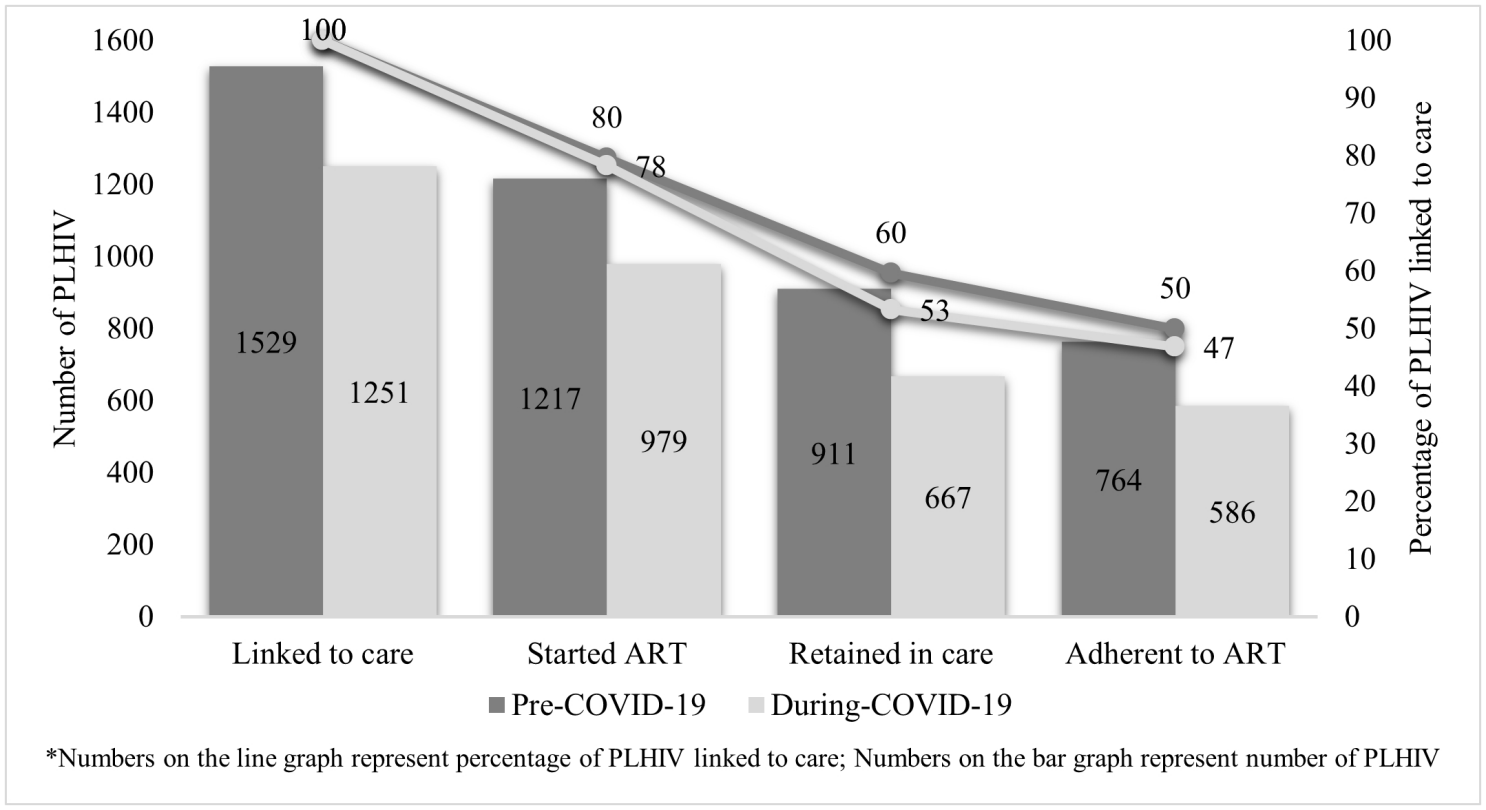
The cascade of HIV treatment in Bandung and Yogyakarta cities.

Compared with the pre-COVID-19 period, a smaller proportion of individuals linked to care were started on ART (79.6% (95%CI 77.5-81.6) vs 78.3% (95%CI 75.9-80.5), p = 0.3892), retained in care (59.6% (95%CI 57.1-62.1) vs 53.3% (95%CI 50.5-56.1), p = 0.0009), and adherent to ART (50.0% (95%CI 47.4-52.5) vs 46.8% (95%CI 44.0-49.7), p = 0.1010) in the COVID-19 period (Fig 1). Results for Bandung and Yogyakarta individually were similar to our pooled results (see Supplementary material, S1 Fig and S2 Fig). Cascade results for *Puskesmas*, private clinic, and hospital can be seen in the Supplementary material file, S3 Fig and S4 Fig.

The multivariate analysis adjusted for PLHIV demographics and clinical characteristics shows that the likelihood of PLHIV being linked to care and starting ART was similar between pre-COVID-19 and during the COVID-19 pandemic (adjusted OR [aOR] = 1.00, 95%CI 0.81-1.23, p = 0.996). Likewise, among PLHIV starting ART, the probability of being retained in care during the pandemic was similar to that in the pre-COVID-19 period (aOR = 0.90, 95%CI 0.73-1.12, p = 0.344). Among those PLHIV on treatment and retained in care, there was a greater adjusted likelihood of being adherent to ART during the COVID-19 pandemic compared to pre-COVID-19 pandemic (aOR = 1.53, 95% CI 1.11 – 2.11, p = 0.009) (Table 2, Fig 1).

**Table 2:** Adjusted* odds of starting ART, being retained in care, and adhering to ART during COVID-19.

COVID-19 era	Started ART (among 2780 linked to care)					Retained in care (among 2196 starting ART)					Adherent to ART (among 1578 retained in care)				
	n(%N)	Univariate OR	p	Multivariate OR*	p	n(%N)	Univariate OR	p	Multivariate OR*	p	n(%N)	Univariate OR	p	Multivariate OR*	p
Pre COVID-19	1,217 (79.6)	1.00		1.00		911 (74.9)	1.00		1.00		764 (83.9)	1.00		1.00	
During COVID-19	979 (78.3)	0.92 (0.77, 1.11)	0.389	1.00 (0.81, 1.23)	0.996	667 (68.1)	0.72 (0.60, 0.87)	0.001	0.90 (0.73, 1.12)	0.344	586 (87.9)	1.39 (1.04, 1.86)	0.026	**1.53 (1.11, 2.11)**	**0.009**

*All odds ratios adjusted for age, sex, education, employment status, marital status, key HIV population, and treatment facility type.

### HIV treatment cascade – subgroup analyses

Full details of our interaction analyses are shown in S1 Table. During the pandemic, those attending hospital facilities were significantly less likely to start ART (aOR = 0.67, 95% CI 0.49 – 0.90), be retained in care (aOR = 0.40, 95% CI 0.27 – 0.59), or remain adherent to ART (aOR = 0.55, 95% CI 0.41 – 0.73) as compared to the pre-COVID-19 period. On the other hand, those attending a *Puskesmas* during the COVID-19 period were significantly more likely to start ART (aOR = 1.41, 95% CI 1.06 – 1.87) and be adherent to ART (aOR = 1.25, 95% CI 1.01 – 1.54) compared to the pre-COVID-19 period. PLHIV aged >40 years were less likely to be retained in care (aOR = 0.47, 95% CI 0.26 – 0.82) or remain adherent to ART (OR = 0.62, 95% CI 0.39 – 0.99) during the COVID-19 pandemic compared with the pre-COVID-19 period. PLHIV aged 20–29 years (OR = 0.71, 95% CI 0.54 – 0.93), men (OR = 0.64, 95% CI 0.51 – 0.79), university or diploma graduates (OR = 0.62, 95% CI 0.41 – 0.93), those employed (OR = 0.72, 95% CI 0.56 – 0.92), those not married (OR = 0.65, 95% CI 0.51 – 0.84), and MSM (OR = 0.76, 95% CI 0.59 – 0.98) were all less likely to be retained in HIV care during the COVID-19 era compared to the pre-COVID-19 era.

## Discussion

Our study shows that, while fewer PLHIV presented for care during the pandemic, HIV treatment cascades in Bandung and Yogyakarta were shown not to be negatively impacted by the COVID-19 pandemic, and that early adherence to HIV treatment actually improved during the pandemic after being adjusted to demographic and clinical characteristics. However, the cascade of care was not consistent across all subgroups evaluated. In particular, the cascade for PLHIV attending hospital services and older PLHIV were negatively impacted by the pandemic.

A reduction in the number of new patients being enrolled in care during the COVID-19 pandemic has been reported in several studies outside of Indonesia [16,17,26]. While some of these reductions may be attributed to decreased risk behaviours such as reduced sexual activity or needle sharing [41,42], in Indonesia, the decline appears to be more strongly linked to structural and socioeconomic barriers. These included fear of contracting COVID-19, mobility restrictions, limited information on ART service availability, requirements for vaccination certificates to travel, and financial hardship [8]. In Yogyakarta and Bandung, city surveillance data and our analysis show that there was an upward trend in HIV cases from 2016 until 2019, followed by a decline in 2020 and 2021, coinciding with the onset of the COVID-19 pandemic [27,30]. In Bandung, new HIV diagnoses dropped from 357 in 2019–82 in 2020 and 43 in 2021, while AIDS diagnoses showed a relatively smaller decrease [43]. Similarly, in Yogyakarta, the number of reported HIV diagnoses decreased from 123 in 2019–36 in 2020, before rising again to 93 in 2023 [44]. A sharp increase in both HIV and AIDS diagnoses in 2023–2024, particularly in Bandung (1,400 new HIV diagnoses and 1,284 AIDS diagnoses in 2024) [43], suggests a rebound in diagnoses as health services resumed and surveillance activities intensified. It is possible that COVID-19 delayed HIV diagnosis in some individuals which reduced transmission rates and led to more PLHIV seeking care at an advanced stage of disease [45,46].

This is the first study to assess the impact of the COVID-19 pandemic on HIV treatment using clinical cohort data from South-East Asia. Previous research has highlighted the pandemic’s effects on HIV retention in care and treatment adherence across various settings. For instance, in Uganda, clinic visits dropped by over 50% after national lockdowns [47]. Similarly, studies from ten countries in Asia [25], the United States [48,49], and Ethiopia [50] recorded a decrease in clinic visits and retention in care, and an increase in lost-to-follow-up cases due to the pandemic. Interruptions to in-person visits and medical follow-up services have also led to lower treatment adherence among PLHIV in several settings [51]. Our unadjusted findings concur with these earlier studies. However, after adjusting for patient and facility characteristics, which these earlier studies did not do, we found that the COVID-19 period was not associated with significant changes to the proportion of PLHIV starting ART or retained in care. Moreover, HIV treatment adherence was actually enhanced in the COVID-19 period in both Bandung and Yogyakarta. This could be attributed to the implementation of various mitigation strategies aimed at managing healthcare services amid the pandemic including multi-month ART dispensing and telemedicine [10]. Additionally, during the pandemic outreach workers pivoted their activities towards virtual outreach while peer support workers delivered medication to the homes of PLHIV [10,23].

It is important to note however that for certain groups, such as those attending hospital services and older PLHIV, the cascade of care was negatively impacted by the pandemic. One possible explanation is that the preparedness of hospitals for delivering services during the pandemic was highly variable, with a national survey showing that preparedness ranged from ‘adequate’ (highest) in 73% of hospitals to ‘not ready’ (lowest) in 9% of hospitals [52]. The Special Region of Yogyakarta demonstrated ‘adequate’ hospital preparedness, whereas in West Java, preparedness ranged from ‘adequate’ to ‘moderate’, with one hospital being classified as ‘not ready’ [52]. Additionally, PLHIV accessing treatment at a one-stop testing and treatment facility has a higher likelihoodin ART commencement and retention following an HIV diagnosis [35,40]. Loss to follow-up was also found to be higher in hospitals than in *Puskesmas* [53].

Furthermore, PLHIV aged ≥55 years were found to be significant predictors for missed appointments during the COVID-19 pandemic [54], suggesting that age-related concerns about infection risk may have adversely affected treatment continuity [55]. In our study, several other subgroups (e.g., men, university or diploma graduates) also had a low likelihood of being retained in HIV care during the COVID-19 era. Retention in HIV care is known to be worse among men than women in Indonesia [56]. In our study, university or diploma graduates were less likely to be retained in HIV care, a finding that contrasts with pre-pandemic research [40]. Many students who come from outside Bandung or Yogyakarta choose to return to their hometowns, of which potentially disrupting their HIV care. While there is limited direct evidence on student migration and its effect on HIV care, widespread internal migration during the pandemic has been reported across Indonesia [57]. This discrepancy underscores the need for further investigation to better understand why HIV care was negatively impacted by the pandemic in each of the subpopulations highlighted in our analysis.

These findings highlight the importance of targeted interventions to address the unique challenges facing the delivery and uptake of HIV services during public health crises and emergencies. Measures employed in Indonesia included testing clients for COVID-19 before providing medical care, enforcing physical distancing at healthcare facilities, revising medicine dispensing schedules, and involving community health workers, community pharmacies, and peer support workers in outreach activities like the home delivery of medicines [23,58,59]. Additionally, telemedicine was utilised for consultations [10]. Many of these mitigation strategies are shown to have improved adherence to ART treatment across a range of LMIC during the pandemic [59–63]. While multi-month ART dispensing has continued in many LMIC [64], it was only a temporary measure in Indonesia and is yet to be widely implemented [65] largely due to weak supply chain systems for ARTs [66,67]. Other strategies such as adjusting the national insurance policy to cover telemedicine and policy support to minimise disruptions in the ARV supply chain should also be explored. Further research is needed to assess the feasibility, effectiveness, and cost-effectiveness of these different strategies, considering the specific needs of PLHIV in Indonesia.

To our knowledge, this is the first retrospective cohort study using clinical cohort data to investigate the impact of the COVID-19 pandemic on the continuity of HIV care in Indonesia and South-East Asia more widely. What sets this research apart is the use of a large four-year cohort data set that focuses on newly diagnosed PLHIV (linked to care) as the starting point for evaluating treatment progression, instead of the more common approach of focussing on PLHIV who are already receiving treatment. However, our study also has some limitations. Mostly public HIV treatment centres in two cities were included, thereby limiting the generalizability of our findings in Indonesia. Our analysis was also based on secondary data generated during the routine clinical management of HIV-positive individuals at participating facilities and was therefore subject to incompleteness. Nonetheless, we were able to address many of the missing data points through extensive consultation and data confirmation with the City Health Office and participating health facilities. Our study did not differentiate between the various waves of the pandemic and their impact on the cascade of care, potentially overlooking useful lessons for policy-making including the timing and design of mitigation strategies.

## Conclusion

The COVID-19 period was associated with a reduction in the number of PLHIV linked to care and disruptions to the HIV treatment cascade among various subpopulations in Bandung and Yogyakarta. These associations may delay progress by the Indonesian government towards ending AIDS by 2030. Implementing general and targeted mitigation strategies such as multi-month dispensing, telemedicine, and health worker task shifting and training could help alleviate these disruptions and support the continuity of HIV care in the face of possible future health crises.

## Supporting information

S1 Fig The cascade of HIV treatment in Yogyakarta. This figure presents the number and percentage of PLHIV at each step of the HIV treatment cascade in Yogyakarta, including linkage to care, ART initiation, retention, and adherence.
(TIF)

S2 Fig The cascade of HIV treatment in Bandung. This figure shows the number and percentage of PLHIV at each step of the HIV treatment cascade in Bandung, from diagnosis and linkage to ART initiation, retention, and adherence.
(TIF)

S3 Fig Number of PLHIV across the HIV treatment cascade by type of health facility. This figure displays the number of PLHIV at each cascade stage, stratified by type of treatment facility.
(TIF)

S4 Fig Percentage of PLHIV across the HIV treatment cascade by type of health facility. This figure presents the percentage distribution of PLHIV at each cascade stage, stratified by health facilities.
(TIF)

S1 Table Adjusted odds of starting ART, being retained in care, and adhering to ART during COVID-19 by sub-group. This table provides adjusted* odds ratios comparing the COVID-19 period with the pre-COVID-19 period for ART initiation, retention, and adherence. Sub-group analyses include age, sex, education level, employment status, marital status, key population category, and type of treatment facility. *Adjusted for age, sex, education, employment status, marital status, key population category, and treatment facility type. ^Pre-COVID-19 period is the reference group.
(DOCX)
