# Supplementary figures and images for: Assessing the impact of the COVID-19 pandemic on uptake of HIV treatment in Bandung and Yogyakarta, Indonesia: A retrospective cohort study
Source: PLOS Glob Public Health. 2025 Dec 23;5(12):e0005666. doi: 10.1371/journal.pgph.0005666 (PMC12725531; doi:10.1371/journal.pgph.0005666)

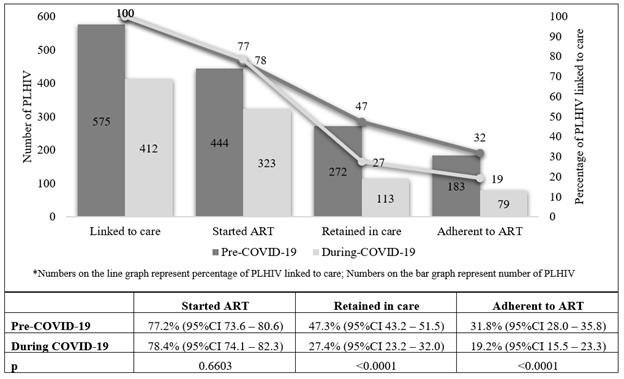

Supplement: S1 Fig — This figure presents the number and percentage of PLHIV at each step of the HIV treatment cascade in Yogyakarta, including linkage to care, ART initiation, retention, and adherence. (TIF) [file pgph.0005666.s001.tif]

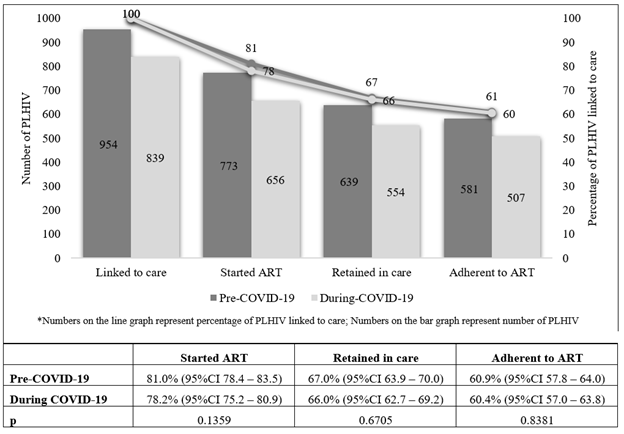

Supplement: S2 Fig — This figure shows the number and percentage of PLHIV at each step of the HIV treatment cascade in Bandung, from diagnosis and linkage to ART initiation, retention, and adherence. (TIF) [file pgph.0005666.s002.tif]

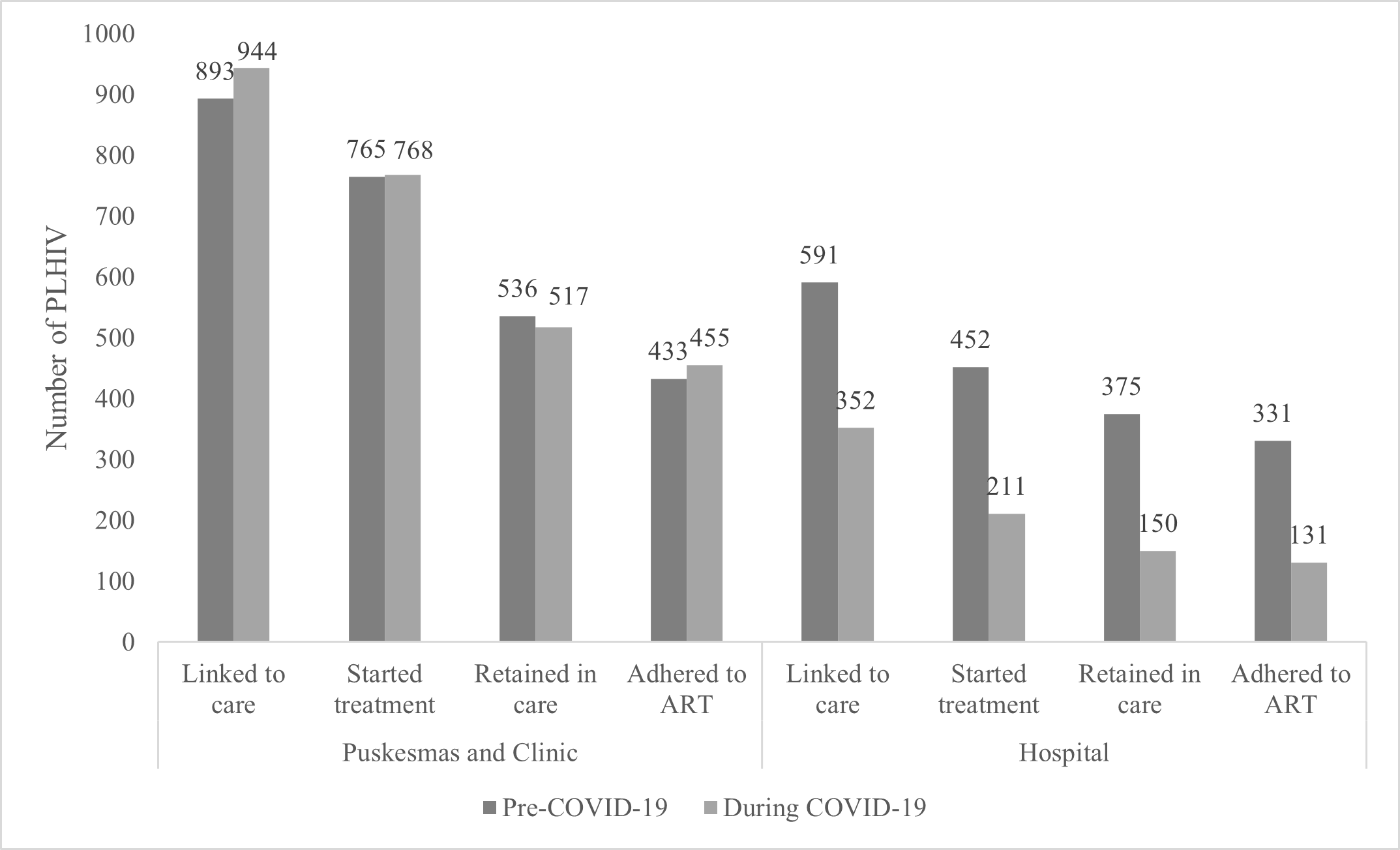

Supplement: S3 Fig — This figure displays the number of PLHIV at each cascade stage, stratified by type of treatment facility. (TIF) [file pgph.0005666.s003.tif]

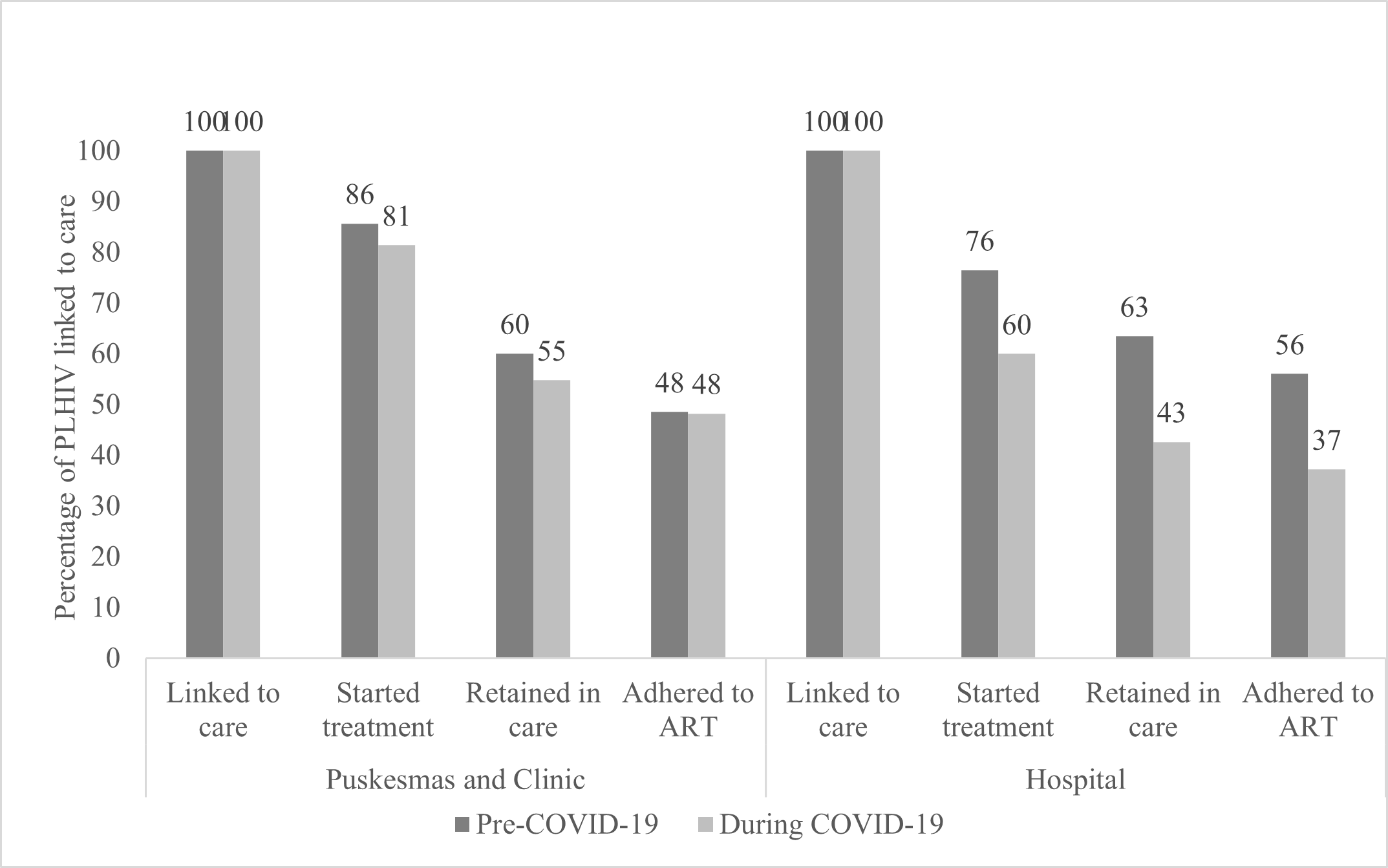

Supplement: S4 Fig — This figure presents the percentage distribution of PLHIV at each cascade stage, stratified by health facilities. (TIF) [file pgph.0005666.s004.tif]
