# Supplementary material for: Assessing the impact of the COVID-19 pandemic on uptake of HIV treatment in Bandung and Yogyakarta, Indonesia: A retrospective cohort study
Source: PLOS Glob Public Health. 2025 Dec 23;5(12):e0005666. doi: 10.1371/journal.pgph.0005666 (PMC12725531; doi:10.1371/journal.pgph.0005666)
Supplement: S1 Table — This table provides adjusted* odds ratios comparing the COVID-19 period with the pre-COVID-19 period for ART initiation, retention, and adherence. Sub-group analyses include age, sex, education level, employment status, marital status, key population category, and type of treatment facility. *Adjusted for age, sex, education, employment status, marital status, key population category, and treatment facility type. ^Pre-COVID-19 period is the reference group. (DOCX) [file pgph.0005666.s005.docx]

**S1 Table. Adjusted* odds of starting ART, being retained in care, and adhering to ART during COVID-19 by sub-group**

| Covariates | **Started ART (Among 2780 linked to care)** | | **Retained in care (Among 2196 starting ART)** | | **Adherent to ART (Among 1578 retained in care)** | |
| --- | --- | --- | --- | --- | --- | --- |
|  | **During COVID-19 versus pre-COVID-19 OR^** | **p** | **During COVID-19 versus pre-COVID-19 OR^** | **p** | **During COVID-19 versus pre-COVID-19 OR^** | **p** |
| **Age group** |  |  |  |  |  |  |
| <20 | 2.88 (0.74, 11.15) | 0.127 | 1.03 (0.38, 2.79) | 0.960 | 1.42 (0.63, 3.18) | 0.397 |
| 20-29 | 1.11 (0.83, 1.49) | 0.467 | **0.71 (0.54, 0.93)** | **0.013** | 1.00 (0.80, 1.25) | 0.975 |
| 30-39 | 0.91 (0.64, 1.30) | 0.600 | 0.68 (0.46, 1.02) | 0.064 | 0.91 (0.66, 1.24) | 0.532 |
| >40 | 0.77 (0.48, 1.26) | 0.300 | **0.47 (0.26, 0.82)** | **0.008** | **0.62 (0.39, 0.99)** | **0.044** |
| **Sex** |  |  |  |  |  |  |
| Male | 0·99 (0.79, 1.24) | 0.924 | **0.64 (0.51, 0.79)** | **<0.001** | 0.91 (0.76, 1.09) | 0.315 |
| Female | 1.06 (0.62, 1.82) | 0.819 | 1.09 (0.58, 2.03) | 0.793 | 1.07 (0.66, 1.76) | 0.775 |
| **Highest level of education** |  |  |  |  |  |  |
| Primary school or less | 0.80 (0.25, 2.58) | 0.704 | 0.87 (0.37, 2.05) | 0.745 | 0.99 (0.44, 2.25) | 0.985 |
| High school | 0.88 (0.67, 1.15) | 0.347 | 0.85 (0.66, 1.10) | 0.226 | 1.06 (0.86, 1.30) | 0.602 |
| University or diploma | 0.85 (0.56, 1.29) | 0.433 | **0.62 (0.41, 0.93)** | **0.022** | 0.87 (0.63, 1.20) | 0.394 |
| **Employment** |  |  |  |  |  |  |
| Employed | 0.96 (0.74, 1.24) | 0.764 | **0.72 (0.56, 0.92)** | **0.009** | 0.99 (0.82, 1.20) | 0.933 |
| Unemployed or student | 0.64 (0.42, 0.99) | 0.043 | 0.95 (0.61, 1.50) | 0.839 | 0.97 (0.68, 1.38) | 0.866 |
| **Marital status** |  |  |  |  |  |  |
| Married | 0.82 (0.54, 1.23) | 0.330 | 0.79 (0.47, 1.31) | 0.356 | 0.85 (0.58, 1.25) | 0.405 |
| Not Married | 1.05 (0.79, 1.38) | 0.750 | **0.65 (0.51, 0.84)** | **0.001** | 0.96 (0.78, 1.17) | 0.673 |
| Divorced | 0.48 (0.16, 1.43) | 0.189 | 1.44 (0.57, 3.62) | 0.439 | 1.04 (0.49, 2.20) | 0.928 |
| **Population group** |  |  |  |  |  |  |
| MSM | 0.99 (0.73, 1.35) | 0.965 | **0.76 (0.59, 0.98)** | **0.033** | 1.12 (0.91, 1.37) | 0.284 |
| FSWs | 0.74 (0.40, 1.35) | 0.323 | 1.09 (0.55, 2.17) | 0.807 | 0.86 (0.51, 1.47) | 0.589 |
| PWID | 2.21 (0.81, 6.03) | 0.122 | 2.57 (0.56, 11.68) | 0.223 | 2.13 (0.61, 7.40) | 0.236 |
| Non-key population/Transgender Women/Waria/Unknown | 1.00 (0.73, 1.38) | 0.980 | **0.37 (0.24, 0.56)** | **<0.001** | **0.51 (0.36, 0.73)** | **<0.001** |
| **Treatment health facilities** |  |  |  |  |  |  |
| Puskesmas and Clinic | **1.41 (1.06, 1.87)** | **0.017** | 0.82 (0.65, 1.05) | 0.123 | **1.25 (1.01, 1.54)** | **0.039** |
| Hospital | **0.67 (0.49, 0.90)** | **0.008** | **0.40 (0.27, 0.59)** | **<0.001** | **0.55 (0.41, 0.73)** | **<0.001** |

*All odds ratios adjusted for age, sex, education, employment status, marital status, key HIV population, and treatment facility type

^Pre-COVID-19 period is the reference group
